# High-Intensity Red Light-Emitting Diode Irradiation Suppresses the Inflammatory Response of Human Periodontal Ligament Stem Cells by Promoting Intracellular ATP Synthesis

**DOI:** 10.3390/life12050736

**Published:** 2022-05-15

**Authors:** Nobuhiro Yamauchi, Emika Minagawa, Kazutaka Imai, Kenjiro Kobuchi, Runbo Li, Yoichiro Taguchi, Makoto Umeda

**Affiliations:** Department of Periodontology, Osaka Dental University, 8-1, Kuzuhahanazono-cho, Hirakata, Osaka 573-1121, Japan; minagawa-e@cc.osaka-dent.ac.jp (E.M.); imai-k@cc.osaka-dent.ac.jp (K.I.); oduperio@gmail.com (K.K.); li-r@cc.osaka-dent.ac.jp (R.L.); umeda-m@cc.osaka-dent.ac.jp (M.U.)

**Keywords:** photobiomodulation therapy, periodontal ligament stem cells, adenosine triphosphate

## Abstract

Periodontitis is an inflammatory lesion in the periodontal tissue. The behavior of human periodontal ligament stem cells (hPDLSCs), which play an important role in periodontal tissue regeneration, is restricted by the influence of inflammatory mediators. Photobiomodulation therapy exerts anti-inflammatory effects. The purpose of this study was to investigate the effects of light-emitting diode (LED) irradiation on the inflammatory responses of hPDLSCs. The light source was a red LED (peak wavelength: 650 nm), and the total absolute irradiance was 400 mW/cm^2^. The inflammatory response in hPDLSCs is induced by tumor necrosis factor (TNF)-α. Adenosine triphosphate (ATP) levels and pro-inflammatory cytokine (interleukin [IL]-6 and IL-8) production were measured 24 h after LED irradiation, and the effects of potassium cyanide (KCN) were investigated. LED irradiation at 6 J/cm^2^ significantly increased the ATP levels and reduced TNF-α-induced IL-6 and IL-8 production. Furthermore, the inhibitory effect of LED irradiation on the production of pro-inflammatory cytokines was inhibited by KCN treatment. The results of this study showed that high-intensity red LED irradiation suppressed the TNF-α-stimulated pro-inflammatory cytokine production in hPDLSCs by promoting ATP synthesis. These results suggest that high-intensity red LED is a useful tool for periodontal tissue regeneration in chronically inflamed tissues.

## 1. Introduction

Periodontitis is characterized by microbially associated, host-mediated inflammation that results in loss of periodontal attachment by inflammatory mediators [1], leading to the destruction of tooth-supporting structures, alveolar bone loss [2], and, eventually, tooth loss. Gingival biofilms and suspected periodontal pathogens induce a host inflammatory immune responses to produce inflammatory cytokines. The inflammatory reaction progresses and is regulated by the induction of other cytokines via paracrine signaling [3]. Inflammatory cytokines (such as tumor necrosis factor [TNF]-α, interleukin [IL]-1β, IL-6, and IL-8) are involved in periodontal tissue inflammation and destruction, and cytokines produced in inflamed periodontal tissues may gain access to the circulation and, consequently, induce systemic disorders [4,5]. TNF-α is a major cytokine that plays an important role in the inflammatory response [6,7]. Its expression and secretion are increased in chronically inflamed tissues in periodontitis, leading to the induction of inflammatory cytokines, such as IL-6 and IL-8.

Periodontal ligament stem cells (PDLSCs) present in periodontal ligament tissues have high proliferative and osteoblast differentiation abilities, similar to other mesenchymal stem cells (MSCs) [8]. PDLSCs have tissue regeneration capacity and exhibit greater potential to regenerate periodontal tissues than other MSCs, such as bone marrow stromal cells [9]. Therefore, PDLSCs play an important role in periodontal tissue regeneration and periodontal disease treatment. Transplantation of PDLSCs into the site of alveolar bone loss has a beneficial effect on periodontal tissue regeneration [10]. However, inflammatory mediators are involved in periodontitis and tissue remodeling, limiting the behavior of PDLSCs. TNF-α inhibits osteoblast differentiation, alkaline phosphatase activity, and expression of osteoblast differentiation genes (integrin binding sialoprotein and RUNX family transcription factor 2) in PDLSCs [11]. Therefore, it is necessary to investigate the effects of inflammatory mediators on adapting PDLSCs for periodontal treatment.

The light of different wavelengths exerts various biological effects in humans [12,13,14,15,16,17,18,19,20]. Light promotes wound healing [12], pain relief [13], and tissue differentiation [14]. Similarly, it is effective in treating oral mucosal diseases [15], candidiasis [16], and dentin hypersensitivity [17]. These treatments are now known as photobiomodulation (PBM) therapy. PBM is also clinically applied in inflammatory diseases, such as thyroiditis [18], arthritis [19], and muscle damage [20]. The field of PBM has evolved primarily by using low-power lasers as light sources, with light-emitting diodes (LEDs) being the most recently used light sources [21]. We previously reported the promotion of osteogenic differentiation of human PDLSCs [22] and human bone marrow stem cells [23] using red LED as part of the PBM.

PBM acts on mitochondrial respiratory chain complex IV to promote adenosine triphosphate (ATP) synthesis [24,25]. Red and near-infrared rays are mainly used as the light sources in terms of tissue penetration depth. Some studies have suggested the anti-inflammatory effects of low-power lasers and LEDs. Low-power laser irradiation at a wavelength of 660 nm suppresses lipopolysaccharide (LPS)-induced inflammatory reactions in human adipose tissue-derived stem cells [26] and human gingival fibroblast cells [27]. Furthermore, irradiation with a red LED having a wavelength of 625 nm suppresses inflammation in human keratinocyte cells [28]. However, only a few reports have been published on the effects of red LED irradiation on the inflammatory response of PDLSCs induced by TNF-α. Therefore, in this study, we investigated the effects of TNF-α-induced human PDLSCs on inflammatory cytokine production and ATP synthesis using high-intensity red LED.

## 2. Materials and Methods

### 2.1. Human PDLSC Isolation and Culture

PDLSCs were cultured from three females (aged 21–27 years) as described in previous studies [22]. The cells were grown in Dulbecco’s modified Eagle’s medium (Nacalai Tesque, Kyoto, Japan) supplemented with 10% fetal bovine serum (Hyclone, Logan, UT, USA) and antibiotics (100 U/mL penicillin, 100 μg/mL streptomycin, and 25 μg/mL amphotericin B; Nacalai Tesque, Kyoto, Japan) and incubated at 37 °C in 5% CO₂. PDLSCs at passage zero (P0) were seeded and cultured until P3–P6 for use in this experiment. Immunocytochemistry for MSC markers, such as CD34, CD45, CD73, CD90, CD105, STRO-1, and SSEA-4, was used to confirm the cell phenotype. PDLSCs were cultured with antibodies against CD34, CD45, CD73, CD90, CD105, STRO-1, and SSEA-4 (Santa Cruz Biotechnology, Santa Cruz, CA, USA), and the nuclei were counterstained with 4′,6-diamidino-2-phenylindole (Dojindo, Kumamoto, Japan). Images of stained cells were obtained using an all-in-one fluorescence microscope (BZ-9000; Keyence Corp., Osaka, Japan). Human PDLSCs were obtained in accordance with the guidelines of Osaka Dental University for Medical Ethics, and all experiments were approved by the Osaka Dental University Medical Ethics Committee (approval no. 110853). This study was conducted in accordance with the Helsinki Declaration of 1975 as revised in 2013. All participants provided written informed consent to participate in this study, and the study design was approved by the appropriate ethics review board.

### 2.2. Irradiation Procedure

In this study, a red LED (LZ1-00R205 Deep Red LED; LedEngin, Santa Clara, CA, USA) was used, which emits a specific wavelength from 600–700 nm, with a peak wavelength of 650 nm. This light source was used in our previous studies [22,23], with a reference review [29]. The review reports that many wavelengths in the red (600–700 nm) spectral region have shown positive results. The intensity of radiant energy was confirmed using a power meter (Nova II; Ophir, North Andover, MA, USA). The intensity of the light source was 1100 mW/cm^2^. The distance from the LED to the cell layer was 22 mm, and the total absolute irradiance was 400 mW/cm^2^ [30]. This distance was determined based on the heights of the culture plates. The radiant exposure can be calculated by multiplying the intensity by the exposure time. The total radiant exposures were 2, 4, 6, 8, and 10 J/cm^2^ for 5, 10, 15, 20, and 25 s, respectively, with continuous output. All irradiation experiments were performed on a clean bench at room temperature in the dark.

### 2.3. Determination of ATP Levels

PDLSCs were plated in a normal culture medium (100 μL/well) at a density of 2 × 10^4^ cells/mL in a 96-well cell culture plate (Costar Stripwell Plate; Corning, NY, USA). In previous studies [22,31], the cells were seeded in disassembled wells of cell culture plates, and each well was irradiated separately. After 24-h of incubation, irradiation was performed. The cells were allowed to recover for 24 h. ATP levels were detected using the CellTiter-Glo 2.0 luminescent cell viability assay (Promega, Madison, WI, USA), following the manufacturer’s protocol. Luminescence was recorded using a multimicrotiter reader (SoftMax Pro Microplate Data Acquisition and Analysis, Molecular Devices; Sunnyvale, CA, USA).

### 2.4. Cell Cytotoxicity Assay

PDLSCs were seeded in a 96-well microplate at a density of 2 × 10^4^ cells/mL in 100 μL of normal culture medium. After 24-h incubation, irradiation was performed. After 24-h, lactate dehydrogenase (LDH) activity was measured. Triton (0.2%; Sigma-Aldrich, St. Louis, MO, USA) was used as a positive control. LDH activity was measured using the Cell Cytotoxicity LDH Assay Kit-WST (Dojindo Laboratory, Kumamoto, Japan), following the manufacturer’s instructions.

### 2.5. Measurements of IL-6 and IL-8 Levels

PDLSCs were plated in normal culture medium at a density of 2 × 10^4^ cells/mL in a 24-well cell culture plate. After 24-h of incubation, the medium was replaced with a medium containing TNF-α (0, 1, 10, or 20 ng/mL), and the cells were cultured for 24 h. The levels of IL-6 and IL-8 in the supernatant were measured using ELISA kits (Thermo Fisher Scientific, Waltham, MA, USA), according to the manufacturer’s instructions.

### 2.6. Potassium Cyanide (KCN) Inactivation of Cytochrome C Oxidase

ATP synthesis was inhibited using KCN (Wako Pure Chemical Industries, Osaka, Japan), an inhibitor of cytochrome C oxidase [32], which is a respiratory chain complex IV within the mitochondria of eukaryotes. Immediately before LED irradiation, the medium was replaced with medium containing KCN (0, 10, 50, 100, 250, or 500 μM).

### 2.7. Statistical Analyses

Statistical analyses were performed using IBM SPSS Statistics ver. 17 (IBM, Chicago, IL, USA). All data are presented as the mean ± standard deviation. One-way analysis of variance followed by Tukey’s test was used to determine statistical significance. *p* values < 0.05 were considered to be statistically significant.

## 3. Results

### 3.1. Isolation and Characterization of PDLSCs

**Figure 1:**
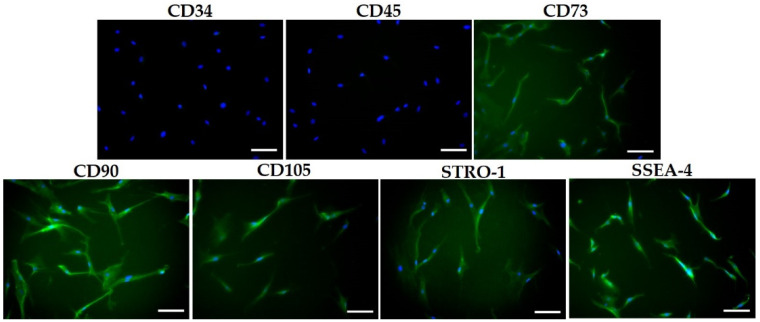
Characterization of human periodontal ligament stem cells (PDLSCs) as mesenchymal stem cells (MSCs). Immunocytochemical staining for CD34, CD45, CD73, CD90, CD105, STRO-1, and SSEA-4. PDLSCs were negative for CD34 and CD45. The PDLSCs were positive for CD73, CD90, CD105, STRO-1, and SSEA-4 (green). Scale bar = 100 μm.

PDLSCs were negative for CD34 and CD45, and positive for CD73, CD90, CD105, STRO-1, and SSEA-4 (Figure 1). MSC must express CD73, CD90 and CD105, and lack expression of CD34 and CD45 [33]. STRO-1 is one of the most well-known markers of MSCs and has been heavily relied upon for the recognition and isolation of various types of MSCs, particularly those in dental tissues [34]. SSEA-4 is an embryonic stem cell marker that is detected in periodontal ligament-derived MSCs [35].

### 3.2. ATP Levels and LDH Activity in PDLSCs after LED Irradiation

**Figure 2:**
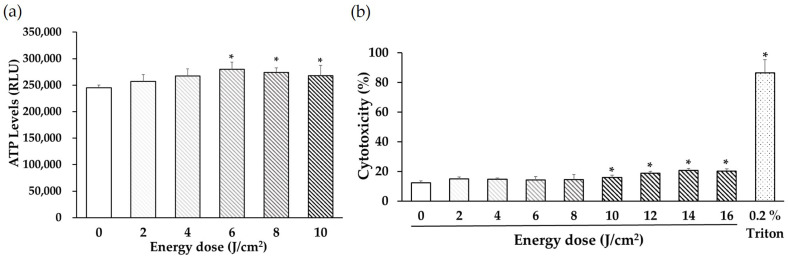
Effects of light-emitting diode (LED) irradiation on adenosine triphosphate (ATP) levels and cytotoxicity in human PDLSCs. (**a**) Effects of energy density of 0–10 J/cm^2^ on PDLSC ATP levels after culture for 24 h. ATP levels were significantly elevated following 6 J/cm^2^ LED irradiation. (**b**) Cytotoxicity was determined after 24 h. Cytotoxicity was significantly increased by irradiation with 10 J/cm^2^ or more. RLU = relative light unit (* *p* < 0.05 vs. control).

To provide optimal stimulation, the effects of energy densities (doses) ranging from 0 to 10 J/cm^2^ on the ATP levels of PDLSCs were examined after culturing for 24 h. LED irradiation induced the highest ATP levels at 6 J/cm^2^ (Figure 2a). LDH activity was measured after 24 h and was found to be significantly increased by irradiation at 10 J/cm^2^ or higher (Figure 2b). A dose of 6 J/cm^2^ was chosen for subsequent assays.

### 3.3. Optimal TNF-α Concentration

**Figure 3:**
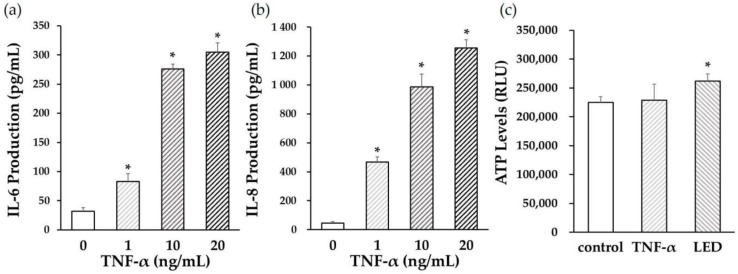
Effects of tumor necrosis factor (TNF)-α on interleukin (IL)-6 and IL-8 production and ATP levels in PDLSCs. IL-6 (**a**) and IL-8 (**b**) production from PDLSCs after 24 h was significantly elevated when TNF-α levels higher than 1 ng/mL were used. (**c**) Effects of TNF-α (1 ng/mL) and LED irradiation (6 J/cm^2^) on ATP levels in PDLSCs after 24 h. TNF-α did not affect the ATP levels in PDLSCs. RLU = relative light unit (* *p* < 0.05, vs. control).

To understand whether TNF-α stimulation affected the pro-inflammatory cytokine production in PDLSCs, we measured the levels of IL-6, IL-8, and TNF-α (1, 10, or 20 ng/mL), after culturing the cells for 24 h. IL-6 (Figure 3a) and IL-8 (Figure 3b) production was significantly elevated when the concentration of TNF-α was higher than 1 ng/mL. These results indicated that treatment with TNF-α at a concentration of 1 ng/mL was sufficient to stimulate the production of inflammatory cytokines in PDLSCs. Additionally, TNF-α at 1 ng/mL did not affect the ATP levels in PDLSCs (Figure 3c).

### 3.4. Production of Pro-Inflammatory Cytokines Stimulated by TNF-α and LED Irradiation

**Figure 4:**
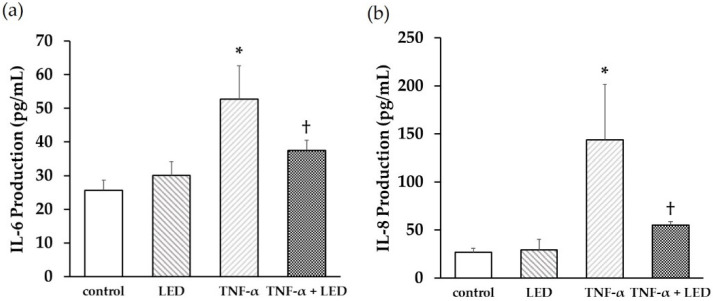
Effects of LED irradiation on TNF-α-stimulated IL-6 and IL-8 production. IL-6 (**a**) and IL-8 (**b**) production significantly increased in the culture medium after 24 h of TNF-α (1 ng/mL) treatment, while co-treatment with LED irradiation (6 J/cm^2^) and TNF-α significantly suppressed this production. (* *p* < 0.05 vs. control, ^†^ *p* < 0.05 vs. TNF-α).

PDLSCs were treated with or without TNF-α (1 ng/mL), immediately followed by LED irradiation at an energy density of 6 J/cm^2^ to examine the effect of LED irradiation on TNF-α-induced inflammatory responses in PDLSCs. The concentrations of IL-6 (Figure 4a) and IL-8 (Figure 4b) in the culture medium increased after 24 h of TNF-α treatment. Co-treatment with LED irradiation and TNF-α induction significantly suppressed IL-6 and IL-8 production (Figure 4a,b).

### 3.5. ATP Synthesis Inhibition by KCN

**Figure 5:**
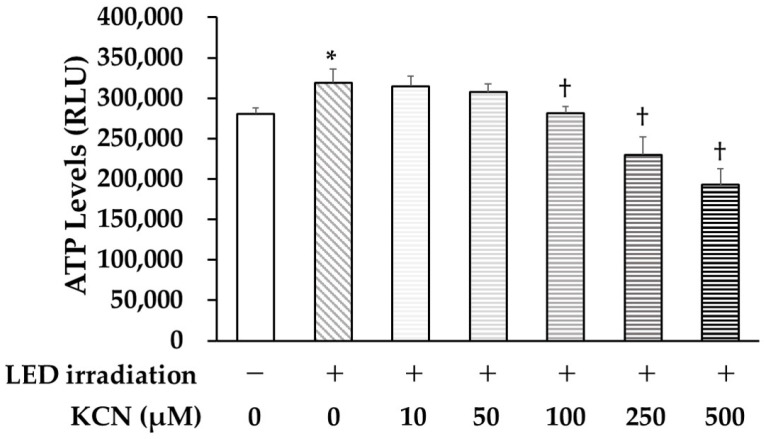
Effect of potassium cyanide (KCN) on LED-induced ATP levels in PDLSCs. Treatment with KCN (>100 μM), ATP synthesis inhibitor, significantly decreased the LED-induced ATP levels in PDLSCs. RLU = relative light unit (* *p* < 0.05 vs. control, ^†^ *p* < 0.05 vs. LED irradiation).

To investigate the effects of LED irradiation on ATP synthesis, irradiated PDLSCs were cultured in a normal medium with KCN. We found that KCN treatment significantly decreased LED-induced ATP levels relative to those in the untreated cells and that treatment with 100 μM KCN blocked the effect of LED irradiation (Figure 5).

### 3.6. KCN Inhibited LED-Induced Anti-Inflammatory Activity

**Figure 6:**
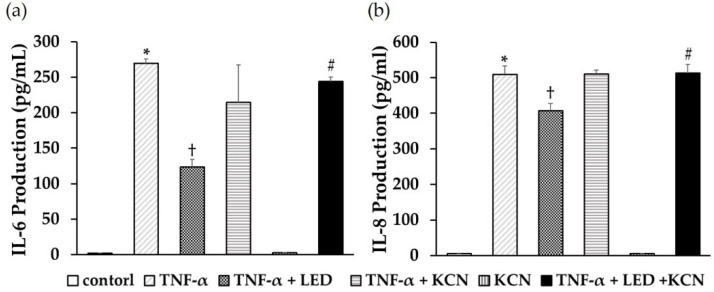
Effects of KCN on suppression of LED-induced IL-6 and IL-8 production in PDLSCs. IL-6 (**a**) and IL-8 (**b**) production from PDLSCs after 24 h under LED irradiation (6 J/cm^2^) after treatment with TNF-α (1 ng/mL) and KCN (100 μM) was significantly increased compared to that in the cells without KCN (* *p* < 0.05 vs. control, ^†^ *p* < 0.05 vs. LED irradiation, ^#^ *p* < 0.05 vs. TNF-α + LED irradiation).

To evaluate the effects of intracellular ATP levels on LED-induced anti-inflammatory activity, we measured IL-6 and IL-8 production from PDLSCs 24 h after LED irradiation (6 J/cm^2^) and treatment with TNF-α (1 ng/mL) and KCN (100 μM). KCN did not promote IL-6 and IL-8 production in PDLSCs and had no effect on TNF-α-induced production. In contrast, KCN significantly increased TNF-α-stimulated IL-6 (Figure 6a) and IL-8 (Figure 6b) production after 24 h of LED irradiation.

## 4. Discussion

The results of this study indicated that high-power red LED irradiation suppressed the inflammatory reaction of PDLSCs induced by TNF-α. We also found that the increase in intracellular ATP levels induced by LED irradiation contributed to the upward control of the anti-inflammatory effects.

The light source for PBM was developed using a laser. However, in recent years, LEDs have attracted attention as light sources. Lasers are highly coherent light sources, whereas LEDs are incoherent in nature. LEDs are inexpensive, safe to use, and do not require any large equipment. The differences between coherent and incoherent PBM effects are being investigated in various ongoing studies. Previous studies suggest the possibility of replacing lasers with LEDs as the light source without significantly affecting the results [21]. Although there are a few reports on the effects of red LED irradiation on PDLSCs, previous studies have reported that PBM activates cytochrome C oxidase, an enzyme within the mitochondria of eukaryotes [24]. This enables the resumption of the respiratory chain activity and ATP synthesis [25]. In the past, Holder et al. [36] reported that 653 nm red LED irradiation increased the intracellular ATP levels of dental pulp cells. Wang et al. [37] reported that the intracellular ATP levels in human adipose-derived stem cells were increased after irradiation with 3 J/cm^2^ of red (660 nm) and near-infrared (810 nm) light sources, but decreased after irradiation with blue (415 nm) and green (540 nm) light sources. Similarly, in this study, ATP levels were increased by red LED irradiation, suggesting that ATP is photoactive. By using a light source with a high output of 400 mW/cm^2^, the ATP levels increase significantly after 15 s of irradiation, and the optimum amount of energy is obtained. In PBM, it is important to determine the optimal irradiation parameters for each specific tissue and surgical procedure because phototherapy success is dictated by several factors, such as wavelength, energy density/dose, and power density of the light source [38,39,40].

PBM exerts anti-inflammatory effects. Sakurai et al. [27] showed that low-power laser irradiation at a wavelength of 660 nm inhibited prostaglandin E2 production by LPS in human gingival fibroblast cells, leading to low cyclooxygenase-2 mRNA levels. Sun et al. [28] also used a red LED with a wavelength of 625 nm to demonstrate the anti-inflammatory effects of human keratinocytes via sphingosine kinase 1 (SPHK1). TNF-α is an inflammatory cytokine that plays a central role in the inflammatory response and is mainly secreted by immune cells, such as macrophages and neutrophils. It induces inflammation by acting on other cells and upregulates the production of pro-inflammatory innate immune cytokines, such as IL-6 and IL-8 [6]. Yamaura et al. [41] reported that PBM was applied to TNF-α-stimulated synoviocytes to reduce IL-1β and IL-8 production. In this study, the production of IL-6 and IL-8 was significantly reduced by irradiating PDLSCs with TNF-α and high-power red LED. This result indicates that high-power red LED exerts anti-inflammatory effects in TNF-α-induced PDLSCs.

ATP maintains cellular function as a phosphorylation substrate inside the cell and functions as an intercellular signal transduction substance outside the cell. Intracellular ATP is involved in the activation of cyclic AMP (cAMP), a major secondary messenger. Intracellular cAMP levels are increased by PBM [42]. cAMP also exerts anti-inflammatory effects [43], and Wu et al. [26] reported that low-power laser irradiation at a wavelength of 660 nm increases intracellular levels of cAMP and suppresses the inflammatory response in LPS-induced human adipose-derived stem cells. As mentioned earlier, PBM has an intracellular ATP synthesis-promoting effect, while TNF-α inhibits intracellular ATP synthesis [44]. Therefore, it is necessary to investigate the effects of intracellular ATP levels on the anti-inflammatory effects of PBM. To investigate the effect of increasing intracellular ATP concentration on the anti-inflammatory effect of red LED irradiation in PDLSCs, the cells were treated with KCN, an ATP synthesis inhibitor. The effect of TNF-α on the production of inflammatory cytokines was observed. KCN is an inhibitor of the mitochondrial respiratory chain complex IV [32], which is associated with PBM and suppresses the production of ATP via oxidative phosphorylation. In this study, KCN alone did not promote IL-6 and IL-8 production in PDLSCs and had no effect on TNF-α-induced IL production. However, KCN inhibited the inhibitory effect of LED irradiation on TNF-α-induced IL-6 and IL-8 production and significantly increased their production. Therefore, promotion of ATP synthesis may be an important factor involved in the suppressive effect of LED irradiation on TNF-α-induced inflammation in PDLSCs.

PDLSCs promote intracellular ATP synthesis by adjusting the amount of energy of high-power red LED irradiation, which further decreases the TNF-α-induced production of pro-inflammatory cytokines. Periodontal tissue is constantly in a chronic inflammatory state owing to the presence of periodontopathic bacteria. Therefore, it is clinically important to regulate the secretion of inflammatory cytokines in PDLSCs, which are essential for periodontal tissue regeneration. Our results suggest that red LED may be useful for periodontal tissue regeneration.

## 5. Conclusions

Our results suggest that PBM therapy with LED can potentially be applied in anti-inflammatory therapy followed by periodontal tissue regeneration. The exact cellular signaling pathways responsible for this anti-inflammatory action are not yet completely understood, and there is a lot of scope for further work on PBM and inflammation. PBM, which has been developed using lasers, can be more cost-effective and widely applied to periodontal treatment by using LED, which is inexpensive, safe to use, and does not require any large equipment.

## Data Availability

Not applicable.
